# Internet and Health Information Technology Use and Psychological Distress Among Older Adults With Self-Reported Vision Impairment: Case-Control Study

**DOI:** 10.2196/17294

**Published:** 2020-06-03

**Authors:** Namkee G Choi, Diana M DiNitto, Othelia EunKyoung Lee, Bryan Y Choi

**Affiliations:** 1 Steve Hicks School of Social Work The University of Texas at Austin Austin, TX United States; 2 School of Social Work University of North Carolina at Charlotte Charlotte, NC United States; 3 Department of Emergency Medicine Brown University Providence, RI United States

**Keywords:** older adults, vision impairment, HIT, psychological distress, digital divide, mobile phone

## Abstract

**Background:**

The number of older adults with vision impairment (VI) is growing. As health care services increasingly call for patients to use technology, it is important to examine internet/health information technology (HIT) use among older adults with VI.

**Objective:**

This study aimed to examine (1) the rates of internet/HIT use among older adults with VI compared with a matched sample of their peers without VI, (2) associations of VI with internet/HIT use, and (3) association of HIT use with psychological distress, assessed with the Kessler-6 screen.

**Methods:**

Data were obtained from the 2013 to 2018 US National Health Interview Survey. Older adults (aged ≥65 years) with self-reported VI were matched with older adults without VI, in a 1:1 ratio, based on age, sex, number of chronic medical conditions, and functional limitations (N=2866). Descriptive statistics and multivariable logistic regression models, with sociodemographic factors, health conditions, health insurance type, and health care service use as covariates, were used to examine the research questions.

**Results:**

In total, 3.28% of older adults (compared with 0.84% of those aged 18-64 years) reported VI, and 25.7% of them were aged ≥85 years. Those with VI were significantly more socioeconomically disadvantaged than those without VI and less likely to use the internet (adjusted odds ratio [aOR] 0.64, 95% CI0.49-0.83) and HIT (aOR 0.74, 95% CI 0.56-0.97). However, among internet users, VI was not associated with HIT use. HIT use was associated with lower odds of mild/moderate or serious psychological distress (aOR 0.62, 95% CI 0.43-0.90), whereas VI was associated with greater odds of mild/moderate or serious distress (aOR 1.84, 95% CI 1.36-2.49). Health care provider contacts were also associated with higher odds of internet or HIT use.

**Conclusions:**

Compared with their matched age peers without VI, older adults with VI are less likely to use HIT because they are less likely to use the internet. Socioeconomically disadvantaged older adults experiencing a digital divide need help to access information and communication technologies through a fee waiver or subsidy to cover internet equipment and subscription and ensure continuous connectivity. Older adults with VI who do not know how to use the internet/HIT but want to learn should be provided instruction, with special attention to accessibility features and adaptive devices. Older adults with a low income also need better access to preventive eye care and treatment of VI as well as other health care services.

## Introduction

### Background

A significant portion of older adults in the United States have vision impairment (VI) and associated disability. According to the 2014 American Community Survey, 4.3% (1.1 million) of the civilian, noninstitutionalized population of adults aged 65 to 74 years and 10% (1.9 million) of those aged ≥75 years reported blindness or serious trouble seeing, even when wearing glasses [1]. In the 2014 National Health Interview Survey (NHIS), 13.5% (6.1 million noninstitutionalized adult participants aged ≥65 years, including 350,000 who were blind) reported “any trouble seeing, even when wearing glasses or contact lenses” [1]. VI increases with advancing age, and the rate for the ≥85 age group in the 2014 NHIS was 22.3% [1]. The Centers for Disease Control and Prevention’s (CDC) analysis of the 2016 Behavioral Risk Factor Surveillance System (BRFSS) data also found that 6.6% of noninstitutionalized older adults reported having a vision-related disability, compared with 2.7% in the 18 to 44 age group and 6.1% in the 45 to 64 age group [2]. The BRFSS data showed that regardless of age group, VI (similar to other types of disability) is more prevalent among women than men, racial/ethnic minorities than non-Hispanic whites, and among those with lower income.

VI in older adults is attributable to refractive error (correctable with glasses) and eye disease (often not correctable or requiring surgical/medical interventions: age-related macular degeneration, ocular complications of diabetes mellitus, glaucoma, and age-related cataracts) [3,4]. According to Swenor et al [3], in the United States, uncorrected refractive error accounts for approximately 79% of VI, and cataracts are the most common correctable eye disease, accounting for approximately 50% of VI from eye disease. There are also effective strategies to prevent or delay late-life vision loss and blindness resulting from other defined diseases [4].

VI has a variety of functional consequences, as it can restrict certain activities that require good vision. One such activity may be technology use, including internet and health information technology (HIT) use. The rate of internet use in older adults has steadily risen over the past decade, with 67% using the internet and 51% having a home broadband connection in 2017 [5]. According to the Health Information National Trends Survey data, seeking health information and communicating with physicians on the web were the most significant areas of increase in internet use among older adults between 2003 and 2011/2012 [6]. However, although the digital health technology divide between genders, racial/ethnic groups, rural/urban residents, and those of various health statuses has narrowed over the years, HIT use has remained lower among those in advanced age, racial/ethnic minorities, and those with less education and lower income [6-8]. Lower levels of internet use among disabled older adults with a low income are largely attributable to the lack of financial resources to obtain computers and technology or medical conditions, disabilities, and associated pain that restrict use [9]. Keränen et al [10] also found that physical frailty was associated with lower information and communications technology (ICT) use independent of age, education, and opinions of technology use among older adults. However, studies on the internet and HIT use among older adults in the United States with VI compared with those without VI are not available, indicating a major gap in knowledge regarding VI’s impact on the digital divide.

As health care services, including provider-patient communications, increasingly rely on technology, the use of HIT by older adults has significant implications for the health care system and older adults themselves. HIT may reduce health care spending by providing preventive health promotion information, facilitating communication with health care providers, and improving health care quality and outcomes for older adults [8,11]. Longitudinal data show that HIT use may be associated with fewer physician visits among older adults with certain chronic health conditions (eg, diabetes). However, cross-sectional data show that HIT use is associated with higher health service use (eg, general practitioners, medical specialists, eye doctors, physical therapists/occupational therapists [PT/OT]) [7,12]. The latter may also be positive, as health information seeking and other HIT use behaviors, either encouraged by providers or initiated by the patient, can contribute to increased disease-related knowledge and better adherence to prevention and treatment regimens. Increased HIT use thus has significant potential for health care cost savings and improved health care quality and outcomes, especially among socioeconomically disadvantaged older adults and those with chronic medical conditions and disability.

ICT use may also impact how older adults perceive social support, loneliness, depression, psychological well-being, and quality of life, but findings vary. Some studies show that internet use results in higher levels of social support, life satisfaction and well-being, and reduced loneliness and depression [13-16]. Other studies indicate that variables such as living arrangements, ethnicity, and contact with family mediate these positive relationships and that more frequent use of ICT was associated with more psychological distress and less sense of community among older adults who were lonelier [17-19]. Elliot et al [20] found that although ICT use was not directly related to depressive symptoms or well-being, it acted as a moderator. In essence, functional limitation was a stronger predictor of depressive symptoms for high users, and ill health was a stronger predictor for nonusers or limited users. However, to our knowledge, there is no published study on the association of internet/HIT use with psychological well-being among older adults with VI.

### Study Aims

Given the increasing number of older adults with VI, this case-control study examined (1) rates of internet and HIT use among a nationally representative sample of older adults with VI, compared with a matched sample of their peers without VI, (2) associations between VI and internet and HIT use, and (3) association of HIT use with psychological distress. The study hypotheses were that VI will be associated with lower odds of internet/HIT use (H1), and HIT use will be associated with lower odds of mild/moderate or serious psychological distress, controlling for VI (H2). Other covariates for the multivariable hypotheses testing were sociodemographic factors, health conditions, health insurance type, and health care service use. These findings may help identify VI as a barrier to internet/HIT use among older adults and suggest ways to improve access to technology for older adults with VI.

## Methods

### Data and Sample

Data from the 2013 to 2018 NHIS public-use data files were downloaded from the CDC’s National Center for Health Statistics website. The NHIS is an annual, cross-sectional household survey that is the principal source of information on the health and health care access of the civilian, noninstitutionalized US population [21]. For each sampled household, interviews are conducted (mostly face-to-face) with an adult family member who answers questions about each family member’s demographic and health status characteristics. The NHIS also collects more detailed health and other data from 1 sample adult from each household, which is the primary data source for this study. Combining 6 consecutive years of annual NHIS data resulted in 190,113 sample adult respondents aged 18 to ≥85 years (NHIS public-use data sets do not provide the chronological age of those aged >85 years). Of the 190,113 sample adults, 19.4% (n=48,287) were aged ≥65 years. In this study, we focused on those aged ≥65 years with VI and their matched peers without VI (n=2866).

### Measures

#### Vision Impairment

In the NHIS, respondents were asked about vision (“any trouble seeing, even when wearing glasses or contact lenses” and “blind or unable to see at all”). They were also asked a series of questions about the levels of difficulty (not at all to cannot do it at all) performing physical functions (eg, walking, climbing) and social activities (eg, going out to social events, participating in social activities) without special equipment. Those who reported any difficulty in any of these activities/functions were then asked a series of follow-up questions about whether the difficulty was caused by specific conditions (eg, vision problems, illnesses, injuries). In this study, we refer to older adults who reported that “vision/problem seeing causes difficulty with activities” as those with VI.

#### Internet Use

Respondents were asked if they had used the internet in the past 12 months (yes=1 and no=0).

#### Health Information Technology Use

Respondents were asked if they had (1) looked up health information on the internet, (2) filled a prescription on the internet, (3) scheduled a medical appointment on the internet, and (4) communicated with a health care provider by email in the past 12 months. In this study, HIT use refers to any of these 4 activities.

#### Psychological Distress

This was assessed using the Kessler-6 screen (K6), a global measure of distress that includes depressive- and anxiety-related symptomology over the 4-week period before test administration [22]. The 6 items were how often you felt nervous, restless/fidgety, so depressed that nothing cheered you up, hopeless, worthless, and that everything was an effort (0=none of the time, 1=little of the time, 2=some of the time, 3=most of the time, and 4=all of the time). K6 has reported a sensitivity of 0.36, a specificity of 0.96, and a total classification accuracy of 0.92 in predicting severe mental illness, defined as any Diagnostic and Statistical Manual of Mental Disorders, Fourth Edition (DSM-IV) disorder, other than a substance use disorder, with a Global Assessment of Functioning score less than 60 occurring in the past 12 months [22]. K6 scores of 13 to 24 (ie, serious distress) indicate a probable DSM-IV disorder and significant impairment in functioning, and K6 scores of 8 to 12 (mild/moderate distress) indicate a probable diagnosable mental illness but with less severe impairment in functioning [23]. Cronbach α for the 6 items among the study sample was 0.86.

#### Sociodemographic Factors

Sociodemographic factors are as follows: (1) age (65-74, 75-84, and ≥85 years), (2) gender, (3) race/ethnicity (non-Hispanic white, non-Hispanic black, Hispanic, Asian, and other), (4) marital status (not married vs married), (5) education (bachelor’s degree vs no degree), and (6) ratio of family income to the official US poverty threshold (<200%, 200%-399%, ≥400%, and missing).

#### Health Status

Indicators of health status are as follows: (1) number (0-10) of diagnosed chronic medical conditions (eg, arthritis, asthma, diabetes, hypertension, heart disease, stroke, chronic kidney disease, liver disease, lung disease, and cancer) with which the respondent had ever been diagnosed, and (2) hearing impairment (“hearing problem causes difficulty with activity,” yes=1 and no=0).

#### Health Insurance

Respondents were asked if they had Medicare, Medicaid, private health insurance, and veterans/military insurance coverage (yes=1 and no=0 for each) in the past 12 months.

#### Health Care Use

Respondents were asked if they had in the past 12 months (1) had an overnight hospitalization, (2) received health care 10 or more times, and (3) saw/talked to a general practitioner, eye doctor, medical specialist, PT/OT, or mental health professional (yes=1 and no=0 for each).

### Data Analysis

All analyses were conducted with Stata 15 (Stata Corp)/MP’s svy function to account for the NHIS’s stratified, multistage probability sampling design and to ensure that variance estimates incorporate the full sampling design. All statistics were weighted except for sample sizes. First, using the ccmatch function [24] in Stata, we matched older adults with VI with those without VI in a 1:1 ratio, based on age, sex, number of chronic medical conditions, any other functional limitation than VI, and self-response vs proxy response status. Second, we used chi-square and 2-tailed *t* tests to describe and compare older adults with and without VI on sociodemographic and health statuses, K6 scores, health insurance, and health care use. For K6 scores, we excluded 57 respondents with missing data on 4 or more items but included 6 respondents who had missing data on 1 item and 3 respondents who had missing data on 2 or 3 items after replacing the missing data with the mean of their nonmissing items on the scale for the summed score. Third, we used chi-square tests and 95% CIs to compare older adults with and without VI on their internet and HIT use. Fourth, we tested H1 (association of VI with internet/HIT use) with the study sample using 2 logistic regression models with VI (vs no VI) as the independent variable and internet use (vs nonuse) and any HIT use (vs nonuse) as the dependent variables. We also used logistic regression to examine the association of VI with any HIT use among internet users. Finally, we tested H2 (association of HIT use with psychological distress) using a logistic regression model with any HIT use (vs nonuse) as the independent variable and mild/moderate or severe distress versus no distress (ie, K6 scores 8-24 vs K6 scores 0-7) as the dependent variable. Variance inflation factor diagnostics, using a cutoff of 2.50 [25], showed that multicollinearity among covariates was not a concern. Logistic regression results are presented as adjusted odds ratios (aORs) with 95% CI. Statistical significance was set at a *P*<.05.

## Results

### Study Sample Selection

Of the 48,287 sample adult respondents aged ≥65 years in the 2013 to 2018 NHIS, 16.07% (n=7896, including 398 blind respondents) reported that they had “any trouble seeing, even when wearing glasses or contact lenses.” However, only 18.73% of those who were not blind but had vision problems and 53.15% of blind older adults reported vision-caused difficulty with activities. As a result, 1630 older adults, including 210 blind persons or 3.28% of all NHIS sample adults aged ≥65 years, were deemed to have VI. The 3.28% rate of VI was significantly higher than the 0.84% rate of VI among the NHIS sample adults aged 18 to 64 years. Annual rates of “any trouble seeing” among the age group ≥65 years showed an overall increasing trend over the study period; however, annual VI rates among the age group ≥65 years were not significantly different over the study period.

Of the 1630 older adults with VI, 1598 were matched in a 1:1 ratio with 1598 older adults without VI. After excluding 165 from each group whose interviews were done by proxy (as some questions were not asked of the proxy respondents), the study sample was 2866 older adults (1433 with and 1433 without VI), representing 2.67 million older adults in the United States.

### Sample Characteristics

As Table 1 shows, of the study sample, 39.94%, 34.37%, and 25.68% of the VI group were in the 65-74, 75-84, and ≥85 age groups, respectively. The proportion of those aged ≥85 years in the VI group was much higher than the proportion of the same age group among all NHIS sample adults (11.38%). As the VI and no VI groups were matched for age, sex, and number of medical conditions, these variables were not significantly different between the 2 groups. However, even after matching, compared with the no VI group, the VI group had significantly higher proportions of racial/ethnic minorities, nonmarried individuals, and those with an income less than 200% of the poverty threshold and Medicaid and lower proportions of those with a college degree and private health insurance.

Of the VI group, 88.15% reported that they had VI for more than a year, and 96.99% reported that VI was a chronic condition. The VI group also included more blind and hearing-impaired individuals, with 31.01% (compared with 2.87% in the no VI group) reporting hearing-caused difficulty with activities. Psychological distress was significantly higher among the VI group, with approximately one-fourth of participants reporting mild/moderate or serious psychological distress in the preceding month. The VI and no VI groups did not differ in rates of hospitalization and visits/consultations with health care professionals other than eye doctors. Although the data did not show the exact number of times these older adults saw/talked with health care providers, a higher proportion of the VI group reported receiving health care services ≥10 times, indicating that they were more frequent health care service users than those without VI.

**Table 1:** Sociodemographic, health insurance, and health care use characteristics of the matched sample

Variables		No VI^a^ (n=1433)	VI (n=1433)	*P* value^b^
**Age (years), %**				.67
	65-74	40.34	39.94	
	75-84	32.51	34.37	
	85+	27.15	25.68	
Female, %		59.21	60.43	.62
**Race/ethnicity, %**				<.001
	Non-Hispanic white	78.75	68.79	
	Non-Hispanic black	10.04	11.09	
	Hispanic	6.98	10.88	
	Non-Hispanic Asian	3.09	7.04	
	Other	1.14	2.20	
Married, %		49.12	42.96	.02
College degree, %		23.36	18.72	.02
**Family income: percentage of poverty**				<.001
	<200	32.59	43.66	
	200-399	31.99	25.08	
	≥400	26.66	21.97	
	Missing	8.76	9.29	
**VI duration, %**				N/A^c^
	>3 months	N/A	1.83	
	3-5 months	N/A	0.80	
	6-12 months	N/A	8.04	
	More than a year	N/A	88.15	
	Missing	N/A	1.18	
**VI as a chronic problem, %**				N/A
	Yes	N/A	96.99	
	No	N/A	3.01	
Blind, %		0.38	12.30	<.001
Hearing impairment, %		2.87	31.01	<.001
Number of medical conditions, mean (SE)		3.14 (0.06)	3.12 (0.06)	.77
**Psychological distress (K6^d^ score)^e^**				<.001
	Mean (SE)	2.70 (0.14)	4.53 (0.20)	
	No distress (K6 score=0-7), %	88.21	75.94	
	Mild/moderate distress (K6 score=8-12), %	7.96	14.14	
	Serious distress (K6 score=13-24), %	3.83	9.92	
**Health insurance, %**				
	Medicare	95.48	93.72	.17
	Medicaid	11.08	21.58	<.001
	Private health insurance	44.40	34.70	<.001
	Military health insurance	8.86	10.43	.26
**Health care use in the past 12 months, %**				
	Hospitalized	23.08	23.49	.83
	Received care 10 or more times	27.97	33.47	.02
	Saw/talked to a general practitioner	89.38	87.77	.30
	Saw/talked to an eye doctor	63.09	71.99	<.001
	Saw/talked to a medical specialist	52.58	47.51	.05
	Saw/talked to a physical/occupational therapist	20.36	23.25	.16
	Saw/talked to a mental health professional	5.97	7.88	.14

^a^VI: vision impairment.

^b^Probability values for differences between the no VI and VI groups were calculated using Pearson chi-square tests for categorical variables and, two-tailed, independent sample *t* tests for continuous variables (number of medical conditions and K6 scores).

^c^N/A: not applicable.

^d^K-6: Kessler-6 screen.

^e^Sample size is 2809 (1413 without VI and 1396 with VI) because of missing data.

### Internet and Health Information Technology Use

Table 2 shows that compared with 42.61% and 30.87% of the no VI group, 29.63% and 22.37% of the VI group used the internet and any HIT, respectively. More specifically, the VI group was significantly less likely to have sought health information on the web or communicated with health care providers via email; however, the 2 groups did not differ with respect to filling a prescription and scheduling a medical appointment on the web. In addition to health information seeking, only a small proportion (3.69%-7.96%) of these older adults with or without VI used HIT for other medical/health purposes. Table 2 also shows that of those who used the internet, the VI group and the no VI group did not differ on the rates of HIT use: 67.04% of the no VI group and 69.58% of the VI group used any HIT. Although internet users’ most common HIT use was for health information seeking, between 10.80% and 18.31% also engaged in the other 3 types of HIT use.

**Table 2:** Internet and health information technology use in the past 12 months among the matched sample

Variable		No VI^a^ (n=1433), % (95% CI)	VI (n=1433), % (95% CI)	*P* value^b^
Internet use		42.61 (39.37-45.93)	29.63 (26.50-32.96)	<.001
**HIT^c^ use**		30.87 (27.95-33.94)	22.37 (19.53-25.48)	<.001
	Looked up health information on the internet	28.59 (25.74-31.63)	20.54 (17.83-23.54)	<.001
	Filled a prescription on the internet	6.71 (5.24-8.55)	4.91 (3.67-6.55)	.11
	Scheduled medical appointment on the internet	5.66 (4.18-7.62)	3.69 (2.52-5.38)	.07
	Communicated with health care provider by email	7.96 (6.21-10.13)	5. 45 (4.14-7.13)	.04
**HIT use among internet users^d^**		67.04 (62.32-71.43)	69.58 (63.42-75.11)	.50
	Looked up health information on the internet	61.81 (56.82-66.56)	65.62 (59.44-71.32)	.33
	Filled a prescription on the internet	15.72 (12.37-19.78)	15.54 (11.60-20.51)	.95
	Scheduled medical appointment on the internet	12.86 (9.56-17.08)	10.80 (7.38-15.56)	.46
	Communicated with health care provider by email	18.31 (14.47-22.89)	16.42 (12.43-21.39)	.55

^a^VI: vision impairment.

^b^Probability values for differences between the no VI and VI groups were calculated using Pearson chi-square tests.

^c^HIT: health information technology.

^d^Sample size is 963 (582 without VI and 381 with VI).

### Association of Vision Impairment With Internet and Health Information Technology Use Multivariable Analyses

Table 3 shows that controlling for sociodemographic characteristics, health status, health insurance, and health care service use, VI was still significantly associated with lower odds of internet (aOR 0.64, 95% CI 0.49-0.83) and HIT (aOR 0.74, 95% CI 0.56-0.97) use, although being blind by itself was not a significant factor. In terms of other significant covariates, being in the 2 older age groups, non-Hispanic black or Hispanic, and nonmarried and receiving health care ≥10 times were associated with lower odds of both internet and HIT use, whereas having a college degree, family income ≥200% of the poverty threshold (compared with <200% of the poverty threshold), and veterans/military insurance and seen/talked to a medical specialist and a PT/OT were associated with greater odds of both internet and HIT use. In addition, receiving Medicaid and having seen/talked to a mental health provider were associated with lower odds, and having seen/talked to a general practitioner and an eye doctor was associated with higher odds of internet use but not HIT use.

Table 3 also shows that among internet users, VI was not a significant factor for HIT use. Age group, marital status, education, and health insurance type were not significant. Compared with non-Hispanic white internet users, only Hispanic internet users had lower odds of HIT use. With respect to family income, only those in the *missing* income category had significantly greater odds than those with income <200% of poverty to have used any HIT. Having seen/talked to a medical specialist and a PT/OT was also associated with greater odds of HIT use among internet users.

**Table 3:** Association of vision impairment with internet use and any health information technology use: logistic regression results.

Variable^a^		Internet use vs nonuse, aOR^b^ (95% CI)	Any HIT^c^ use vs nonuse, aOR^b^ (95% CI)	Any HIT use vs nonuse among internet users, aOR^b^ (95% CI)
Vision impairment vs no impairment		0.64 (0.49-0.83)^d^	0.74 (0.56-0.97)^e^	1.15 (0.77-1.74)
Blindness vs no blindness		0.83 (0.44-1.57)	1.08 (0.54-2.17)	2.32 (0.72-7.43)
**Age group (years; vs 65-74 years)**				
	75-84	0.39 (0.29-0.52)^f^	0.45 (0.34-0.59)^f^	0.92 (0.61-1.37)
	≥85	0.20 (0.15-0.27)^f^	0.17 (0.11-0.25)^f^	0.36 (0.20-0.63)
Female vs male		1.31 (1.00-1.71)	1.25 (0.96-1.64)	1.02 (0.69-1.49
**Race/ethnicity (vs non-Hispanic white)**				
	Non-Hispanic black	0.35 (0.23-0.54)^f^	0.44 (0.28-0.71)^d^	0.87 (0.40-1.86)
	Hispanic	0.23 (0.14-0.40)^f^	0.23 (0.12-0.45)^f^	0.37 (0.16-0.83)^e^
	Non-Hispanic Asian	0.53 (0.31-0.91)^e^	0.86 (0.41-1.77)	1.81 (0.50-6.54)
	Other	1.07 (0.53-2.15)	0.89 (0.41-1.94)	0.69 (0.22-2.19)
Not married vs married		0.70 (0.55-0.89)^d^	0.76 (0.59-0.99)^e^	1.00 (0.70-1.43)
College degree vs no degree		4.17 (3.09-5.62)^f^	3.04 (2.23-4.12)^f^	1.27 (0.86-1.87)
**Family income (vs <200% of the poverty threshold)**				
	200-399	1.37 (1.03-1.83)^e^	1.53 (1.11-2.12)^f^	1.23 (0.75-2.04)
	≥400	1.93 (1.40-2.66)^f^	2.01 (1.41-2.86)^g^	1.46 (0.88-2.43)
	Missing	1.01 (0.67-1.53)	1.41 (0.90-2.22)	2.71 (1.11-6.62)^f^
Number of medical conditions		0.96 (0.88-1.03)	1.00 (0.92-1.08)	1.08 (0.96-1.22)
Hearing impairment vs no impairment		1.09 (0.75-1.59)	0.97 (0.66-1.43)	0.95 (0.54-1.69)
Medicaid vs no Medicaid		0.63 (0.41-0.96)^e^	0.76 (0.48-1.19)	1.24 (0.63-2.45)
Private HI^g^ vs no private HI		1.07 (0.83-1.39)	1.15 (0.88-1.52)	1.25 (0.63-2.45)
Military HI vs no military HI		1.58 (1.08-2.31)^e^	1.53 (1.02-2.31)^e^	0.87 (0.47-1.60)
Hospitalized vs not hospitalized		0.92 (0.68-1.25)	0.87 (0.61-1.24)	0.80 (0.51-1.24)
Received care ≥10 times vs received no/less care		0.71 (0.54-0.92)^e^	0.88 (0.65-1.18)	1.22 (0.79-1.88)
Saw/talked to a general practitioner vs did not see/talk		1.80 (1.21-2.67)^d^	1.27 (0.84-1.93)	0.90 (0.35-2.04)
Saw/talked to an eye doctor vs did not see/talk		1.35 (1.04-1.74)^e^	1.31 (0.99-1.74)	1.01 (0.68-1.52)
Saw/talked to a medical specialist vs did not see/talk		1.50 (1.16-1.95)^d^	1.81(1.40-2.35)^f^	1.80 (1.21-2.68)^d^
Saw/talked to a physical/occupational therapist vs did not see/talk		1.41 (1.04-1.91)^e^	1.51 (1.11-2.05)^e^	1.57 (1.00-2.44)^e^
Saw/talked to a mental health professional vs did not see/talk		0.53 (0.33-0.85)^d^	0.86 (0.57-1.30)	1.48 (0.73-3.00)

^a^Model statistics: N=2866, design *df*=885, F (26, 860)=14.54, *P* value <.001 for internet use versus nonuse; N=2866, design *df*=885, F (26, 860)=10.90, *P* value <.001 for any HIT use vs nonuse; and N=963, design *df*=794, F (26, 860)=2.13; *P* value <.001 for any HIT use vs nonuse among internet users.

^b^aOR: adjusted odds ratio.

^c^HIT: health information technology.

^d^*P*<.01.

^e^*P*<.05.

^f^*P*<.001.

^g^HI: health insurance.

### Association of Health Information Technology Use With Psychological Distress: Multivariable Analyses

Table 4 shows that controlling for sociodemographic characteristics, health status, health insurance, and health care service use, HIT use was significantly associated with lower odds of mild/moderate or serious psychological distress (aOR 0.62, 95% CI 0.43-0.90). As expected, VI was associated with greater odds of mild/moderate or serious psychological distress (aOR 1.84, 95% CI 1.36-2.49), but blindness by itself was not. Of the other covariates, being in the 2 older age groups and black, and having a college degree and private health insurance were associated with lower odds of mild/moderate or serious distress; however, being female and nonmarried, having a higher number of medical conditions and hearing impairment, and being seen/having talked to a mental health provider were associated with greater odds of mild/moderate or serious distress. The interaction terms between HIT use and VI were not significant. The findings from the logistic regression model with internet use as the dependent variable were similar, showing lower odds of mild/moderate or serious distress among internet users but higher odds of such distress among those with VI.

**Table 4:** Association of any health information technology use with psychological distress: logistic regression results.

Variable^a^		Mild/moderate or serious vs no distress, aOR^b^ (95% CI)	
Any health information technology use		0.62 (0.43-0.90)^c^	
Vision impairment vs no impairment		1.84 (1.36-2.49)^d^	
Blindness vs no blindness		1.44 (0.83-2.51)	
**Age group (vs 65-74 years)**			
	75-84		0.53 (0.38-0.74)^d^
	≥85		0.46 (0.31-0.70)^d^
Female vs male		1.40 (1.02-1.91)^c^	
**Race/ethnicity (vs non-Hispanic white)**			
	Non-Hispanic black		0.61 (0.39-0.93)^c^
	Hispanic		1.10 (0.68-1.76)
	Non-Hispanic Asian		1.18 (0.61-2.28)
	Other		0.76 (0.37-1.56)
Not married vs married		1.40 (1.04-190)^c^	
College degree vs no degree		0.60 (0.39-0.92)^c^	
**Family income (vs <200% of the poverty threshold)**			
	200-399		1.09 (0.76-1.55)
	≥400+		0.95 (0.65-1.38)
	Missing		0.74 (0.43-1.28)
Number of medical conditions		1.15 (1.05-1.26)^e^	
Hearing impairment vs no impairment		1.90 (1.38-2.61)^d^	
Medicaid vs no Medicaid		1.18 (0.79-1.75)	
Private HI^f^ vs no private HI		0.87 (0.63-1.20)	
Military HI vs no military HI		0.68 (0.41-1.15)	
Hospitalized vs not hospitalized		1.10 (0.78-1.56)	
Received care ≥10 times vs received no/less care		1.30 (0.97-1.74)	
Saw/talked to a general doctor vs did not see/talk		1.40 (0.89-2.20)	
Saw/talked to an eye doctor vs did not see/talk		0.80 (0.59-1.07)	
Saw/talked to a medical specialist vs did not see/talk		0.91 (0.68-1.22)	
Saw/talked to a physical/occupational therapist vs did not see/talk		1.01 (0.73-1.39)	
Saw/talked to a mental health professional vs did not see/talk		3.83 (2.44-5.99)^d^	

^a^Model statistics: N=2809, design *df*=885, F (27, 859)=6.40, *P* value <.001.

^b^aOR: adjusted odds ratio.

^c^*P*<.05.

^d^*P*<.001.

^e^*P*<.01.

^f^HI: health insurance.

## Discussion

### Principal Findings

This study shows that 3.28% of older adults in the United States (compared with 0.84% of those aged 18-64 years) reported that their VI caused difficulty with activities. As expected, the ≥85 age group was overrepresented among those with VI as common age-related eye disorders (eg, macular degeneration, glaucoma, and cataract) tend to be associated with a gradual decline in vision [26]. Although these older adults with VI were matched in a 1:1 ratio with their peers without VI based on age, gender, and health/other disability status, those with VI were still significantly more socioeconomically disadvantaged in terms of education and income, included more racial/ethnic minorities, and had higher proportions of those with blindness and hearing impairment. They also used more health care in the past year.

The first key finding is that older adults with VI are less likely to engage in internet and HIT use than their matched age peers, even after controlling for other sociodemographic, health status, health insurance, and health care use variables, which supports H1. However, among those who used the internet, VI was not associated with the odds of HIT use. This indicates that among older adults with VI, their lower likelihood of internet use is the main reason for their overall lower rate of HIT use compared with their matched age peers. Internet users with VI are likely to navigate the internet using screen magnification and reading software and other accessibility features for vision [27] and are as likely to use HIT as their peers without VI. The second key finding is that HIT use is associated with a lower likelihood of having experienced mild/moderate or serious psychological distress in the past month, controlling for VI, which supports H2. VI is associated with greater odds of having experienced mild/moderate or severe psychological distress, suggesting that VI (and hearing impairment) negatively impacts overall well-being.

The lower odds of internet use among older adults with VI may be because of their lower socioeconomic status and higher levels of health problems and disability that previous studies identified as major contributors to the digital divide [7,8]. Costs of an internet subscription and equipment (eg, computer, tablet) are not likely to be priority items, especially among older adults with a low income who have to contend with the financial strains of managing their health and disability (eg, costs of prescription medications and transportation to health care appointments). Given that almost one-third of older adults with VI also had hearing impairment, these double disabilities were likely barriers to internet and HIT use. A survey of individuals aged 50 to 74 years in the United Kingdom found that those with moderate/serious hearing difficulties were less likely to use computers than those with no hearing difficulty [28].

Our findings also show that internet use was higher if older adults saw/talked to a general practitioner, eye doctor, medical specialist, or PT/OT, and HIT use was higher if they saw/talked to a medical specialist or PT/OT. Those who had visits with an eye doctor may be more socioeconomically advantaged than those who did not. Ehrlich et al [29] found that unmarried, older adults with a low income were less likely than their more advantaged peers to report a recent eye examination, and common reasons for not having an eye examination included cost and lack of insurance coverage. Thus, the relationship between health care provider contact and internet use may reflect the digital divide because of socioeconomic status. The association of HIT use with specialist and PT/OT consultations is also consistent with a previous study [7], suggesting that patients with complex medical conditions that lead to specialist care may be more likely to use HIT to better understand their conditions and treatments. Physical and occupational therapists also tend to spend more time with patients than physician providers do, which allows them more opportunities for patient education. Thus, patient education may have extended the use of web-based resources and encouragement for older adult patients to use these resources as part of promoting specific condition- and treatment-related knowledge as well as overall health to prevent disease, disability, and injury. The importance of integrating health promotion and wellness in PT/OT practice has also been underscored [30].

The finding that internet/HIT use was higher if older adults had veterans/military health insurance regardless of VI is likely because of the fact that the Veterans Administration (VA) health care systems are the most digitalized and telehealth-oriented of all health care systems [31]. VA patients may receive greater encouragement to use HIT to seek health information and navigate the VA health care systems. More research is needed on specific health care service/system use and HIT use among older adults.

Along with the costs of technology adoption and continued connectivity, lack of interest in and distrust of the internet as a source of health information and means of health management may be barriers to the use of HIT by older adults. A survey done in the United Kingdom on the use/nonuse of consumer electronic devices (eg, smartphones, tablet computers, and electronic book [ebook] readers) among people with VI found that cost and lack of interest were among the most frequently cited reasons for nonuse [32]. An earlier study also found that older adults were less likely than middle-aged adults to trust the internet as a source of health information; however, the association between age and distrust was no longer significant after adjusting for potential contributors to distrust, such as confusion in using the internet and providing too much information [33]. Lack of interest may also be caused by a lack of knowledge and skills for adopting technology.

Given the divergent findings discussed earlier on the relationship between ICT use and psychological well-being among older adults, more research is needed to examine moderators and mediators of the association between HIT use and lower likelihood of mild/moderate or serious psychological distress found in the study sample. Among those with VI, ICT use may facilitate social connections (eg, participation in online support and interest groups) and informational/instrumental activities (eg, reading/listening to ebooks, watching/listening to live-streamed religious services). Studies show that ICT use among older adults is associated with increased engagement/participation in social, instrumental, and leisure activities, with some ICT use compensating for aging-related and other challenging circumstances [34,35].

### Limitations

This study has limitations because of data constraints. First, VI was self-reported, and data on age of onset and change over time were not available. Thus, we do not know if some older adults with VI had stopped using the internet because of progressing VI. Second, because the NHIS reports HIT use as a dichotomous variable (use vs nonuse), we could not study the effects of HIT use frequency. More detailed data on HIT use frequency and context can help in better understanding their effects on the well-being of older adults. Third, additional data on the type of activities negatively affected by VI (and other disabilities) would also have been helpful in better understanding their impacts and recommending strategies that can help people with disabilities use the internet and HIT to their advantage. Fourth, because the NHIS data are cross-sectional, we can report associations (correlations) but not causal relationships.

### Conclusions

As more health care services and instrumental daily living activities (eg, banking, paying bills, applying for social services) require ICT use, the digital divide among older adults with VI must be closed. Given the potential of ICT to help older adults with VI remain active and enrich their lives, closing the digital divide may also improve their psychological well-being. First, we recommend that socioeconomically disadvantaged older adults experiencing the digital divide should be provided help to access ICT through a fee waiver or subsidy to cover internet equipment (eg, computer, tablet) and subscription services. The movement for equitable internet access as a public utility has gained momentum in state and local governments [36]. Older adults should be included as targets as they are the largest group of consumers of health care services and services that increasingly rely on ICT use. Second, older adults with VI who do not know how to use the internet and HIT but want to learn should be provided instruction and technology support services. Attention must be paid to accessibility features and adaptive devices that will facilitate technology use among those with VI. Third, in addition to improved ICT access, older adults with a low income need better access to preventive eye care and treatment of VI and other health care services. As refractive errors and some age-related eye diseases can be effectively treated to improve vision, more concerted efforts are needed to reduce VI in late life. Improved access to other health care services and providers is also important for patient education, prevention, treatment, and alleviation of health problems and functional impairment. As our study shows, certain health care provider contacts are associated with higher HIT use among older adults, suggesting attempts to better understand their health problems and treatments using technology.

## Abbreviations

aOR: adjusted odds ratio
BRFSS: Behavioral Risk Factor Surveillance System
CDC: Centers for Disease Control and Prevention
DSM-IV: Diagnostic and Statistical Manual of Mental Disorders, Fourth Edition
ebook: electronic book
HIT: health information technology
ICT: information and communications technology
K6: Kessler-6 screen
NHIS: National Health Interview Survey
PT/OT: physical therapists/occupational therapists
VA: Veterans Administration
VI: vision impairment
